# Nutrient-imbalanced conditions shift the interplay between zooplankton and gut microbiota

**DOI:** 10.1186/s12864-020-07333-z

**Published:** 2021-01-07

**Authors:** Yingdong Li, Zhimeng Xu, Hongbin Liu

**Affiliations:** 1 Department of Ocean Science, The Hong Kong University of Science and Technology, Clear Water Bay, Kowloon, Hong Kong, SAR China; 2 SZU-HKUST Joint PhD Program in Marine Environmental Science, Shenzhen University, Shenzhen, China; 3 Institute for Advanced Study, Shenzhen University, Shenzhen, China; 4 Hong Kong Branch of Southern Marine Science & Engineering Guangdong Laboratory, The Hong Kong University of Science and Technology, Hong Kong, China

## Abstract

**Background:**

Nutrient stoichiometry of phytoplankton frequently changes with aquatic ambient nutrient concentrations, which is mainly influenced by anthropogenic water treatment and the ecosystem dynamics. Consequently, the stoichiometry of phytoplankton can markedly alter the metabolism and growth of zooplankton. However, the effects of nutrient-imbalanced prey on the interplay between zooplankton and their gut microbiota remain unknown. Using metatranscriptome, a 16 s rRNA amplicon-based neutral community model (NCM) and experimental validation, we investigated the interactions between *Daphnia magna* and its gut microbiota in a nutrient-imbalanced algal diet.

**Results:**

Our results showed that in nutrient-depleted water, the nutrient-enriched zooplankton gut stimulated the accumulation of microbial polyphosphate in fecal pellets under phosphorus limitation and the microbial assimilation of ammonia under nitrogen limitation. Compared with the nutrient replete group, both N and P limitation markedly promoted the gene expression of the gut microbiome for organic matter degradation but repressed that for anaerobic metabolisms. In the nutrient limited diet, the gut microbial community exhibited a higher fit to NCM (*R*^*2*^ = 0.624 and 0.781, for N- and P-limitation, respectively) when compared with the Control group (*R*^*2*^ = 0.542), suggesting increased ambient-gut exchange process favored by compensatory feeding. Further, an additional axenic grazing experiment revealed that the growth of *D. magna* can still benefit from gut microbiota under a nutrient-imbalanced diet.

**Conclusions:**

Together, these results demonstrated that under a nutrient-imbalanced diet, the microbes not only benefit themselves by absorbing excess nutrients inside the zooplankton gut but also help zooplankton to survive during nutrient limitation.

**Supplementary Information:**

The online version contains supplementary material available at 10.1186/s12864-020-07333-z.

## Background

The concept of stoichiometric homeostasis is the ability of an organism to maintain its elemental or biochemical composition, despite changes in the quality of resource supply (i.e., food quality) [31, 69]. In aquatic systems, primary producers usually experience dynamic fluctuations in the availability of nutrient resources; therefore, phytoplankton are more flexible in regulating their elemental composition (e.g., C:P, C:N and N:P ratios) than most heterotrophs [22, 23]. For instance, due to the combination of the seasonal variations in Pearl River discharge, strong hydrodynamic mixing of different water masses due to monsoon winds, and inputs of sewage effluent, the effects of interconversion between N and P limitation on the nutrient stoichiometry of phytoplankton was reported (Xu et al. 2008).

In the framework of stoichiometry, prey with a similar elemental ratio as their consumers can enhance the assimilation efficiency of the consumers [69]. However, the highly variable stoichiometry of aquatic primary producers means that herbivorous zooplankton frequently have problems with nutritional imbalance [68]. Numerous studies have been conducted to investigate the effects of nutritionally imbalanced algal food on crustacean mesozooplankton [3, 4]. The results indicate that the elemental composition of primary producers not only affects the growth, grazing behavior, and fecal parameters of herbivorous zooplankton, but it also constrains ecological processes, such as food-web dynamics and the composition of fecal pellets, which are key for nutrient recycling [21, 22]. However, little is known about the effects of the nutrient-imbalanced algal prey on the metabolic interactions between zooplankton and their gut microbes, as well as the properties of the fecal pellets produced by the zooplankton.

Recent studies have revealed that gut microbiota are essential for the survival and environmental adaption of herbivorous zooplankton under various conditions [10, 45]. The dynamic gut microbial community consists of ingested bacteria that pass through the intestinal tract, newly-settled ingested bacteria and the original bacteria [73]. Thus, the environmental conditions can mediate the composition and function by affecting the ambient bacteria that may be ingested by zooplankton and settling in their intestine, resulting in an indirect effect on the growth and fitness of zooplankton. Indeed, the gut microbiota influences nutrient uptake efficiency [9], food digestion rate [9], detoxification of toxic substances [45], and the growth of the *D.magna* [11]. In addition, the dynamic gut microbiota of zooplankton are highly dependent on the ingested ambient bacteria such that although some will be excreted, others will remain and survive [73]. However, it remains unclear how the ingested bacteria react to the transformation in their environment, from the oligotrophic ambient water to the eutrophic zooplankton gut, since the amassed food particles in the latter create a nutrient-rich environment. Since the physiological changes of zooplankton have dramatic effects on global primary production and the nutrient cycle [57, 67], it is therefore important to investigate how zooplankton benefit from the change of metabolic activity of their intestinal microbiota under a nitrogen- or phosphorus-deficient algal diet.

As an important component of global phosphorus cycling, polyphosphate (polyP) is accumulated by microorganisms when the phosphorus concentration is high via luxury uptake and used under phosphorus stress [37, 41]. Although previous studies have demonstrated that accumulation of polyP is common in the gut of insects and is promoted under low-pH conditions, it is still unclear whether polyP will be accumulated in the zooplankton gut and influenced by the stoichiometry changes of prey [17, 50]. Also, there are currently no reports describing how the gut microbiome might affect the biochemical properties of zooplankton fecal pellets, which are one of the main sources of particulate organic carbon that can be exported to the deep ocean [67]. The physical and chemical properties (e.g., the density and organic content) of fecal pellets are strongly influenced by the type, quality, and quantity of the prey and their associated microbes. It is then reasonable to hypothesize that the microbial metabolism in the zooplankton gut plays an important role in mediating the digestibility of the prey and the biodegradability of the fecal pellets, which affects the carbon and nutrient recycling and flux in aquatic ecosystems.

*Daphnia magna*, a widespread freshwater cladoceran with a short maturation period (5–8 days) and strong fecundity (more than 40 eggs every 7 days), is a well-established model zooplankton species for various ecologic and toxicological tests [30, 56]. In the present study, adult *D. magna* was used as the experimental subject and fed with different types of nutrient-imbalanced algal prey. We sequenced the metatranscriptome and 16 s rRNA amplicon of the gut extracted from the *Daphnia magna*, and the life history traits, including clearance rate, ingestion rate, neonates production, and body length were recorded. In this investigation, we aimed to decipher the interdependence and interplay between the host and gut microbiota in a nutrient-imbalanced algal diet. We investigated how microbiota, which were previously subjected to nutrient starvation stress, reacted to the nutrient-enriched *D. magna* intestinal environment; how the host and gut microbiota cooperated in the provision of nutrients; and how the gut microbiota mediated the properties of *D. magna* fecal pellets in a nutrient-imbalanced algal diet.

## Methods

### Preparation of the experimental organisms

The algal prey, *Chlamydomonas reinhardtii* (CC1690), were grown in liquid BG11 medium [61], and *D. magna* were cultured in Aachener Daphnien Medium (ADaM) [36]. Both were cultured in a sterile temperature-controlled chamber at 23 ± 1 °C on a 14:10 h light/dark cycle under 20 μmol m^− 2^ s^− 1^ illumination, with constant stirring and aeration. *D. magna* were kept at a density of one individual per 10 mL and fed with saturating amounts of *C. reinhardtii* (10^5^ cells/mL) each day, and the medium was refreshed once a week. N- and P-limited *C. reinhardtii* cultures were prepared with liquid nitrogen and phosphate-free BG11 medium [61], respectively.

### Grazing experiment

**Fig. 1:**
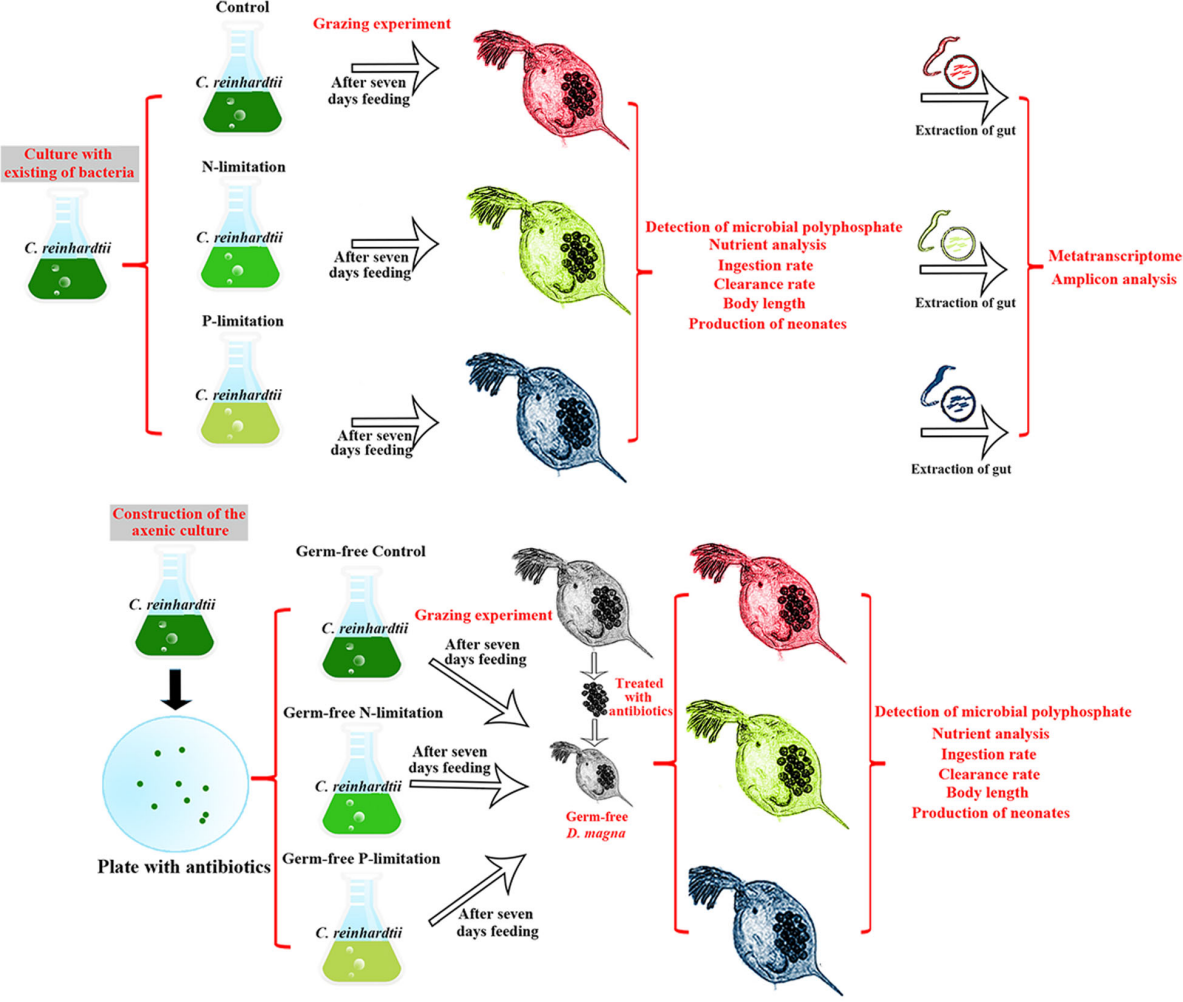
Schematic diagram showing the experimental procedure. The algal prey, *Chlamydomonas reinhardtii* (*C. reinhardtii*), and zooplankton predator, *Daphnia magna* (*D. magna*), is used in this study

Three different *C. reinhardtii* cultures (cultures grown in nutrient-balanced, N-limited or P-limited media) were used to feed the *D. magna* for 7 days (Fig. 1). The prey was centrifuged and re-suspended with an appropriate amount of *D. magna* culture medium before being fed to the *D. magna*. In total, 270 adult *D. magna* were used for each experimental group. Each experimental group consisted of triplicate 1 L PC bottles, each containing 80 adult *D. magna*, incubated in a sterile temperature-controlled chamber as mentioned above. All of these *D. magna* were used for metatranscriptome sequencing. The *D. magna* were kept at a density of one individual per 10 mL (total volume of 800 mL ADaM medium per bottle) and were fed with saturating amounts of nutrient-balanced, N-limited, or P-limited *C. reinhardtii* cells (10^5^ cells/mL) each day throughout the experimental period. For measuring the clearance and ingestion rates, a separate set of triplicate 150 mL PC bottles were prepared for the three experimental groups (nutrient-balanced, N-limited, and P-limited) with 100 mL ADaM medium and 10 *D. magna* in each bottle (a total of 30 individuals were used at the beginning of each experimental group), and the medium and bottles were renewed every day to avoid the influence of any remaining algae in the bottles throughout the experimental period. In these experiments, the neonates were removed from the culture and counted. To avoid cell aggregation or settlement, the cultures were gently agitated manually 2 to 3 times a day. As a control for the grazing experimental groups and to calculate the ingestion rate, another three groups were prepared in triplicate using the same concentration and type of *C. reinhardtii* but no *D. magna*. At the end of the grazing experiment, 20 individuals of *D. magna* from the 150 mL PC bottles in each experimental group were used for body length measurement, and subsequently 16S rRNA amplicon sequencing. The remaining 10 individuals of *D. magna* from the 150 mL PC bottles in each experimental group were used for the determination of the elemental composition. The calculations of ingestion and clearance rate were based on the previously reported method [79]. In brief, Clearance (F, μL Individual^− 1^ d^− 1^) and ingestion (I, cells Individual^− 1^ d^− 1^) rates were calculated according to the following equations, respectively:

1 $$ \mathrm{F}=\ln \left({\mathrm{C}}_{\mathrm{t}}^{\prime }/{\mathrm{C}}_{\mathrm{t}}\right)\times \left(\mathrm{V}/\mathrm{nt}\right) $$

2 $$ \mathrm{I}=\mathrm{F}\times \left[\mathrm{C}\right] $$

Within eq. (1), C_t_′ and C_t_ (cells mL − 1) stand for the prey concentrations at the end of the incubation in control and experimental bottles, respectively; V is the volume of the culture (mL); t (d) is the incubation period, and n is the number of *D. magna* used. For eq. (2), [C] is the prey concentration in the experimental bottle averaged over the incubation period.

### Flow cytometry analysis

To determine the bacterial cell abundance inside the liquid algal cultures, filtrate samples were collected from the three different experimental groups before and after the grazing experiment via filtration through a 1 μm-pore-size filter. The filtrate samples were then stained with SYBR Green I solution at a ratio of 10:1 (the SYBR Green I solution was 1:1000 diluted with Milli-Q water; Molecular Probes) and incubated at 37 °C in the dark for 1 h [48]. The bacterial cell abundance was then examined using the Becton-Dickson FACSCalibur flow cytometer.

### Construction of the axenic culture

In a series of experiments (Fig. 1), sterile cultures of *C. reinhardtii* and *D. magna* were established using antibiotics, as described in previous studies [34, 45]. For the establishment of sterile *C. reinhardtii* culture, R medium containing a cocktail of antibiotics (ampicillin in 500 μg/mL, carbendazim in 100 μg/mL, and cefotaxime in 40 μg/mL (Sigma, Germany)) was used to obtain a pure *C. reinhardtii* colony. As ampicillin and carbendazim can be heat-inactivated, they were added to the agar medium after it was autoclaved and immediately before the plates were poured. Carbendazim was added to the agar medium before it was autoclaved and then the solution was mixed well before the plates were poured, as it is heat stable but only barely soluble [34]. After inoculating *C. reinhardtii* to the plate and 14 days of cultivation in the sterile temperature-controlled chamber (23 ± 1 °C on a 14:10 h light/dark cycle), the pure algal colonies were obtained and then inoculated into the autoclaved liquid BG11 medium. The remaining bacterial abundance in the culture was examined with a Becton-Dickson FACSCalibur flow cytometer.

For the construction of the axenic zooplankton culture, the eggs of *D. magna* from the control group were treated with antibiotics, hatched in a sterile environment, and fed with axenic *C. reinhardtii* cells*.* In brief*,* bacteria-free eggs were obtained by disinfecting eggs, from the normally fed *D. magna*, through exposing them to 0.25% ampicillin (Sigma, Germany) for 30 mins. A part of the antibiotic-treated eggs was crushed with a pestle and filtered through 0.22 μm membrane for PCR assessment of remaining bacteria [43]. After rinsing with sterile ADaM to remove ampicillin, the eggs were transferred to a sterile six-well plate for hatching. The axenic grazing experiment was conducted in triplicate in 150 mL PC bottles and incubated in the sterile temperature-controlled chamber mentioned above with 10 *D. magna* inside each bottle, where the axenic *C. reinhardtii* was used as prey*.* At the end of the grazing experiment, all the survived *D. magna* (in total 30 individuals were used at the beginning of each experimental group) in each experimental group (Germ-free Control, Germ-free N-limited, and Germ-free P-limited) were used for the measurement of body length.

### Nutrient analyses

Before the beginning of the grazing experiment, samples of *C. reinhardtii* that had been grown in different conditions were collected for the analysis of cellular carbon, nitrogen, and phosphorus. Samples were taken from the respective culture bottles by filtering 15 to 25 mL of each culture onto pre-combusted (i.e., at 550 °C for 5 h) GF/C glass-fiber filters. After the seven-day grazing experiment or following 6-h starvation, five individuals of *D. magna* from each experimental group were transferred to a pre-combusted 25 μm GF/C filter for determination of elemental composition (C and N), and another five individuals of *D. magna* of similar body length and weight as the first five were collected for phosphorus measurement. Cellular carbon and nitrogen in both the *D. magna* and *C. reinhardtii* were measured with a CHNS (carbon, hydrogen, nitrogen, sulphur) elemental analyzer (FlashSmart CHNS, Thermo Scientific Inc. Massachusetts, USA) according to previously described protocol [78]. The amount of phosphorus (in the form of orthophosphate) was analyzed manually following acidic oxidative hydrolysis with 1% HCl [25] using a spectrophotometer at a wavelength of 880 nm, with a detection limit of 0.5 μmol/L.

### Gut extraction of *D. magna*

For the molecular investigation, triplicate 1 L PC bottles were prepared for the three experimental groups (nutrient-balanced, N-limited, and P-limited) with 80 individuals raised in each bottle. At the end of the seven-day grazing experiment, 260 guts of *D. magna* from each experimental group were extracted, including 240 guts from triplicate 1 L PC bottles and 20 guts from triplicate 150 mL PC bottles mentioned previously). The gut was extracted with sterilized (i.e., autoclaved and 70% ethanol steeped) dissection tweezers (Regine 5, Switzerland) in a sterile Petri dish under a stereomicroscope (see Video 1). Before each gut extraction procedure, tweezers were flame-sterilized and rinsed with 70% alcohol. Each of the extracted guts from the various experimental groups was placed into a 1.5 mL sterile Eppendorf tube and dissociated into a cell suspension according to the previous report [42]. The cell suspension was then filtered through a 0.22 μm polycarbonate membrane (EMD Millipore, Billerica, MA, USA) with the addition of 500 μL RNA protect reagent (Qiagen, Germany). To assess the potential operation contamination, the tweezers and Petri dishes used to prepare the cell suspension were rinsed with water and this was then filtered through another 0.22 μm membrane for the detection of contamination. In total, 18 filters were used to collect the cell suspension from the gut and the contamination separately. All the filters were preserved in sterile 1.5 mL Eppendorf tubes and stored at − 80 °C until RNA extraction.

### Detection of microbial polyphosphate

Ten adult *D. magna* from each experimental group (i.e., nutrient-balanced, N-limited, or P-limited) were placed in 100 mL of sterile ADaM medium to empty their guts, and their fecal pellets were collected by filtering the medium through a 2.0 μm polycarbonate membrane (EMD Millipore, Billerica, MA, USA). The membrane was sonicated for 30 s to release any bacteria that were attached to the fecal pellets into the suspension. The fecal detritus was removed via centrifugation at 4000 g for 5 mins, and the supernatant was used for the detection of microbial polyP. To detect microbial polyP in zooplankton and algal culture, the culture was firstly filtered through a 3 μm membrane to remove the algal and large particles. Then the filtrate was used for the detection of microbial polyP according to a previous report [38]. In brief, the released cells (in a 96-well plate) were stained with 25 mM Tris/HCl at pH 7.0 containing 500 μg/mL DAPI for 10 min, and the level of fluorescence was measured using a Flex Station 3 multimode microplate reader with excitation and emission filters of 420 nm and 550 nm, respectively (Molecular Devices, Sunnyvale, CA, USA). The microbial protein was then further quantified as described previously [2], and the fluorescence intensity of microbial polyP was expressed as relative fluorescence units (r.f.u.) per mg of total cellular protein.

### DNA extraction and PCR amplification of 16S rRNA gene

The investigation of bacterial contaminant and gut microbial community variation was achieved through DNA extraction and PCR amplification of the 16S rRNA gene. Total genomic DNA was extracted from the filters of dissection tools rinsed with bacteria-free water and from randomly sampled *D. magna* germ-free eggs using a PureLink Genomic DNA kit (Invitrogen, ThermoFisher Scientific Corp., Carlsbad, CA, USA). The extracted DNA was then eluted into 100 μl Tris-EDTA (TE) buffer for PCR amplification. Due to occasional failures of gut extraction, a different number of *D. magna* guts were collected from the Control (10), N-limitation (7) and P-limitation (12) experimental groups. Each of these guts was placed into tubes individually for amplification of the 16S rRNA gene. These 29 gut microbial communities were amplified and sequenced as described previously [44]. In brief, 16 s rRNA gene was amplified with the forward primer 341F (5′-CCTACGGGRSGCAGCAG-3′) and reverse primer 787R (5′-CTACNRGGGTATCTAA-3′). The cycling conditions were as follows: predenaturing at 95 °C for 5 min; 30 cycles of denaturing at 95 °C for 45 s, annealing at 55 °C for 45 s, extension at 72 °C for 60 s; and a final extension at 72 °C for 10 min. The PCR reactions were conducted in triplicates, and the products were pooled together and sequenced by a Hiseq 2500 System (Illumina, San Diego, CA, USA) with 2× 250 bp paired-end read configurations.

### Analysis of 16S rRNA gene

The sequenced contig reads between 135 and 152 bp were preserved, and primers as well as low-quality reads were removed with FASTX-Toolkit [54]. Reads with an average Phred score < 25 were discarded, as were reads with any consecutive runs of low-quality bases > 3. The lowest quality score allowed was 3, the minimum of continuous high-quality bases was 75% of the whole read length, and the maximum number of ambiguous bases was 0 [52]. Chimeras were identified and removed using UCHIME [19]. The remaining high-quality sequences were merged using cat command in the Linux system according to the experimental treatments, and the taxonomic assignment was processed with the Silva database (version 123) using the qiime2 affiliated feature-classifier command [5]. Finally, sequences were clustered into OTUs with a 97% sequence similarity cutoff. To get an overall gut community distribution pattern within each experimental treatment, the OTUs were normalized with the sample number before further analyses. The results were further used in an LDA (linear discriminant analysis) effective size (LEfSe) analysis, which is commonly used to reveal the microbial community differences between experimental groups. In general, the LDA score is calculated from the comparison between two groups, and a higher absolute value of LDA indicates that the species is more enriched in one group.

### RNA isolation and metatranscriptomic sequencing

The filters collected during the various experiments were briefly thawed on ice and the RNA protection solution was removed as previously described [76]. In brief, the filters were transferred to a new 0.7-ml tube with a pinhole at the bottom. This was placed on top of a 1.5-ml centrifuge tube, and the residual RNA protection reagent was removed from the filters when the two tubes were centrifuged at 1000 rpm for 1 min. RNA extraction was achieved with the Totally RNA isolation kit (Ambion Inc., Germany) according to the manufacturer’s protocol. The Turbo DNA-free DNase kit (Ambion Inc., Germany) was used to remove the remaining DNA, then a Nanodrop spectrophotometer (Nanodrop Technologies, Wilmington, USA) was used to examine the purity of the extracted RNA. The RNA BR Assay kit (Life Technologies, Invitrogen, Germany) in conjunction with a Qubit® 2.0 flurometer was utilized to estimate the concentration. The sequencing library was prepared using the NEBNext Ultra Directional RNA Library Prep Kit for Illumina (NEB) following the manufacturer’s recommendations [28]. The pooled RNA from each triplicate was barcoded and sequenced with an Illumina HiSeq2500 sequencer (Novogene Co., Ltd., China), generating between 131.3 and 207.1 million 150 bp paired-end reads per replicate.

### Disentangling partner reads from the holobiont system

In total, nine samples including triplicate Control, N-limitation, and P-limitation were used for metatranscriptome sequencing. According to the barcode, the sequencing data were assigned to nine experimental groups (Control, N-limitation and P-limitation). The quality control of sequenced reads was performed as described in previous reports [24, 53]. In addition, the reads that belong to different parts of the holobiont (i.e., *D. magna* and its gut microbiota) were separated by applying a previously reported method [49]. In brief, the genome and previously published RNA-seq datasets of *D. magna* [51] were downloaded to a local server to construct a host reference library, and the bacterial fractions of the Tara Oceans meta-genomic gene catalogue (OM-RGC) and non-redundant (nr) database were extracted with the blastdbcmd program [12] to build a microbiota reference library. The SRC_c software [47] was then used to map the metatranscriptomic data either to the host or to the gut microbiota with indexed k-mers set to 32 and suggested default similarity s value (50%).

### Reads assembly and downstream analysis

After separation of the *D. magna* and gut microbiota affiliated metatranscriptomic data, the reads were assembled into longer transcripts, separately, using Trans-ABySS v2.0.1 [62] with multiple k-mer sizes from 32 to 92 and a step of 4. Transdecoder (v5.3.0) [26] was used to predict the open reading frames (ORFs) of the assembly result (The ORFs is the mRNA region of the assembly result). The annotation of ORFs was achieved using DIAMOND (v0.9.21.122) [7] against the Kyoto Encyclopedia of Genes and Genomes (KEGG) database and the nr database, with the following parameters: blastp; k parameter = 1; and an e-value = 10^− 7^. For calculation of the coverage information of ORFs, reads were mapped back to the ORFs using Bowtie 2.2.9 [39] and SAMtools v1.9 [40]. The differentially expressed genes (DEGs) between experimental groups were calculated according to a previous report [42], using the edgeR package in R [63]. The samples of triplicate control and N-limitation were used in control vs. N-limitation, while samples in triplicate control and P-limitation were used in control vs. P-limitation. The DEGs were defined with the criteria of |log_2_ (fold change)| > 1 and *p*-value < 0.05 shown in the comparisons between experimental groups. Additionally, the genes encoding microbial butyrate synthesis were also identified using the specific database [74].

### Gene expression validation

To validate the RNA sequencing results, six microbial genes and seven *D. magna* genes that are known to be involved in important biological functions were selected for further validation via an RT-qPCR approach. For each sample, HiScript® III RT SuperMix for qPCR (+ gDNA wiper) (Vazyme Biotech, Nanjing, China) was used for the reverse transcription of extracted DNA-free RNA (500 ng). Reverse transcription (RT) control of each pair of primers was also used in the qPCR experiment for the detection of the possible remaining DNA in the extracted RNA. After the synthesis of cDNA, 1 μL (47 ng) from each cDNA sample was used for qPCR with a Fast start Universal SYBR Green Master mix kit (Roche, Germany) in a LightCycler 384 device (Roche, Germany). The thermocycling conditions were as follows: an initial hold at 50 °C for 2 min and at 95 °C for 10 min followed by 45 cycles of 95 °C for 15 s and 60 °C for 1 min. All reactions were performed in triplicate. The relative amount of mRNA was determined using the 2^−ΔΔCt^ method, and the 16S rRNA gene was selected as a reference for normalization of the gut microbe genes. The primers used to target specific genes in the gut microbiota and *D. magna* were as previously described [42] and they are listed in Table S1.

### Statistical analyses

For the ingestion rate, reproduction, and final body length, data were presented as the mean ± SD derived from the biological replicates. Student’s *t*-tests (two-tailed) were conducted with significance levels of *p* < 0.05. Similar to previous calculation about the neutral processes in the gut microbial community of zebrafish over host development [8], Sloan’s neutral community model (NCM) was constructed to evaluate the contribution of neutral processes in *D. magna*’s gut community structure under different diets [65]. The analysis was performed with R 3.6.1 statistical software. In this analysis, Nm is an estimate of dispersal between communities while the R^2^ determines the overall fit to the neutral community model [14]. Canonical correspondence analysis (CCA) was performed using the PAST 3.0 software.

## Results

### Construction of axenic cultures

Axenic *C. reinhardtii* cells were obtained from agar plates containing an antibiotic cocktail comprising ampicillin (500 μg/mL), carbendazim (40 μg/mL), and cefotaxime (100 μg/mL). It was apparent that after 14 days in cultivation, the antibiotics markedly inhibited the growth of other microorganisms (Fig. S1B), including prokaryotes and fungus when compared with the antibiotic-absent control group (Fig. S1A). After the inoculation of the axenic *C. reinhardtii* cells from the agar plate to sterile liquid media, the bacterial abundance was measured before and after the grazing experiment by flow cytometry. Since the detected bacterial abundance in all liquid algal cultures was extremely low (< 5 cells/μL, Table S2), their impact on the results of the feeding experiments was negligible (Table S2). The 16S amplicon results obtained for the antibiotic-treated eggs, and the extracted gut of *D. magna* after being fed with different types of sterile algal prey, showed that there was no PCR product band in the gel, which confirmed that the *D. magna* were successfully manipulated into axenic conditions. In addition, without intestinal bacteria, the mean body length (0.51 and 0.53 mm for P-limitation and N-limitation, respectively) and survival rate (averaged 12 and 11% for P-limitation and N-limitation, respectively) of *D. magna* were both lower than these parameters in the Control group (0.77 mm of body length, and 23% survival rate). Furthermore, in the sterile P- and N-limited groups, the values of these life-history traits (body length and mortality rate) were not only lower than they were in the sterile Control group, but also lower than that in the germy P- and N-limited groups after 7 days of feeding (Fig. S2A & B).

### Elemental composition of *C. reinhardtii* and *D. magna*

Manipulation of nutrients in the media produced *C. reinhardtii* cells with different elemental compositions*.* The N- or P-limited medium resulted in lower amounts of cellular N or P, respectively, when compared with their nutrient-balanced counterparts (Table 1). Accordingly, *C. reinhardtii* cells showed the highest molar C:N ratio when cultured in N-limited medium, whereas the highest molar C:P ratio was detected in cells cultured in P-limited medium (Table 1). As *C. reinhardtii* is a source of food for *D. magna,* the distinctively different nutritional quality of these preys markedly affects the elemental composition of the predator. Thus, measurements of the elemental composition of the *D. magna* indicated that the highest molar C:N and C:P ratios were detected in the cultures fed with N- and P-limited prey, respectively, regardless of whether the experimental group was germ-free or not.

**Table 1:** Summary of elemental composition of *C. reinhardtii* and *D. magna*

Experimental groups	*C. reinhardtii*		*D. magna*	
	C:N (Mean ± SD)	C:P (Mean ± SD)	C:N (Mean ± SD)	C:P (Mean ± SD)
Control	7.5 ± 0.2	65.0 ± 6.1	12.1 ± 1.0	19.1 ± 0.7
N-limitation	16.7 ± 0.1	74.3 ± 8.7	16.0 ± 1.4	20.9 ± 1.2
P-limitation	15.4 ± 0.3	467.7 ± 89.8	20.9 ± 1.2	22.3 ± 1.0
Germ-free Control	6.7 ± 0.4	57.4 ± 5.8	15.1 ± 0.7	20.0 ± 0.8
Germ-free N-limitation	14.5 ± 0.5	67.1 ± 4.6	17.4 ± 1.1	21.1 ± 1.3
Germ-free P-limitation	13.7 ± 0.7	551.4 ± 71.4	21.1 ± 0.6	22.1 ± 0.8

*Mean* mean value for different elemental ratio

*SD* standard deviation

### Effects of low-quality prey on the life history traits of the *D. magna*

**Fig. 2:**
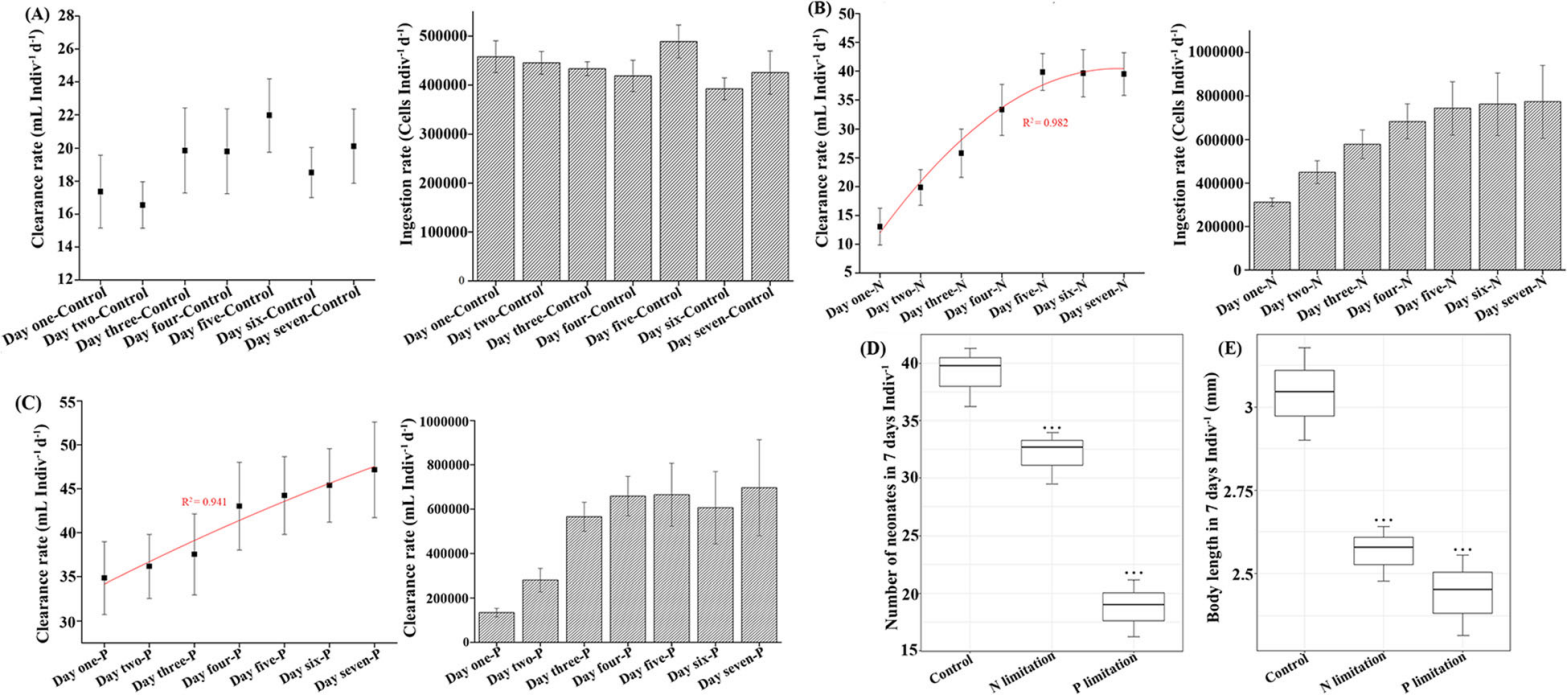
**a** *D. magna* related clearance and ingestion rate during 7 days under nutrient balanced condition. The clearance and ingestion rate of *Daphnia magna* (*D. magna*) during 7 days under (**b**) N- and (**c**) P-limitation. **d** The number of neonates generated by each *D. magna* over a period of 7 days, and (**e**) the body length of *D. magna* at the end of 7 days following the different experimental treatments shown. Error bars in the graphs are ± standard deviation calculated for data from the triplicate experiments. *** denotes statistically significant (*P* < 0.05) lower values in number of neonates and body length than those in the controls

The life-history traits of the *D. magna* were markedly affected by the nutritional quality of their prey (Fig. 2). For example, the ingestion and clearance rates of *D. magna* were found to increase in the poor-quality diet when compared with the Control group (Fig. 2a, b, c). The results also showed that the ingestion and clearance rates of the *D. magna* continuously increased with the length of time they were fed on low-quality prey, although in the P-limited group, the rates plateaued at day six. In addition, the t-test showed that when compared with the Control group, both the number of neonates (Fig. 2d) and body length (Fig. 2e) of *D. magna* significantly decreased when they were fed poor-quality prey (*P* < 0.05), with more severe effects found in the P-limited diet.

### Disentanglement of the partner transcriptome in the holobiont

**Fig. 3:**
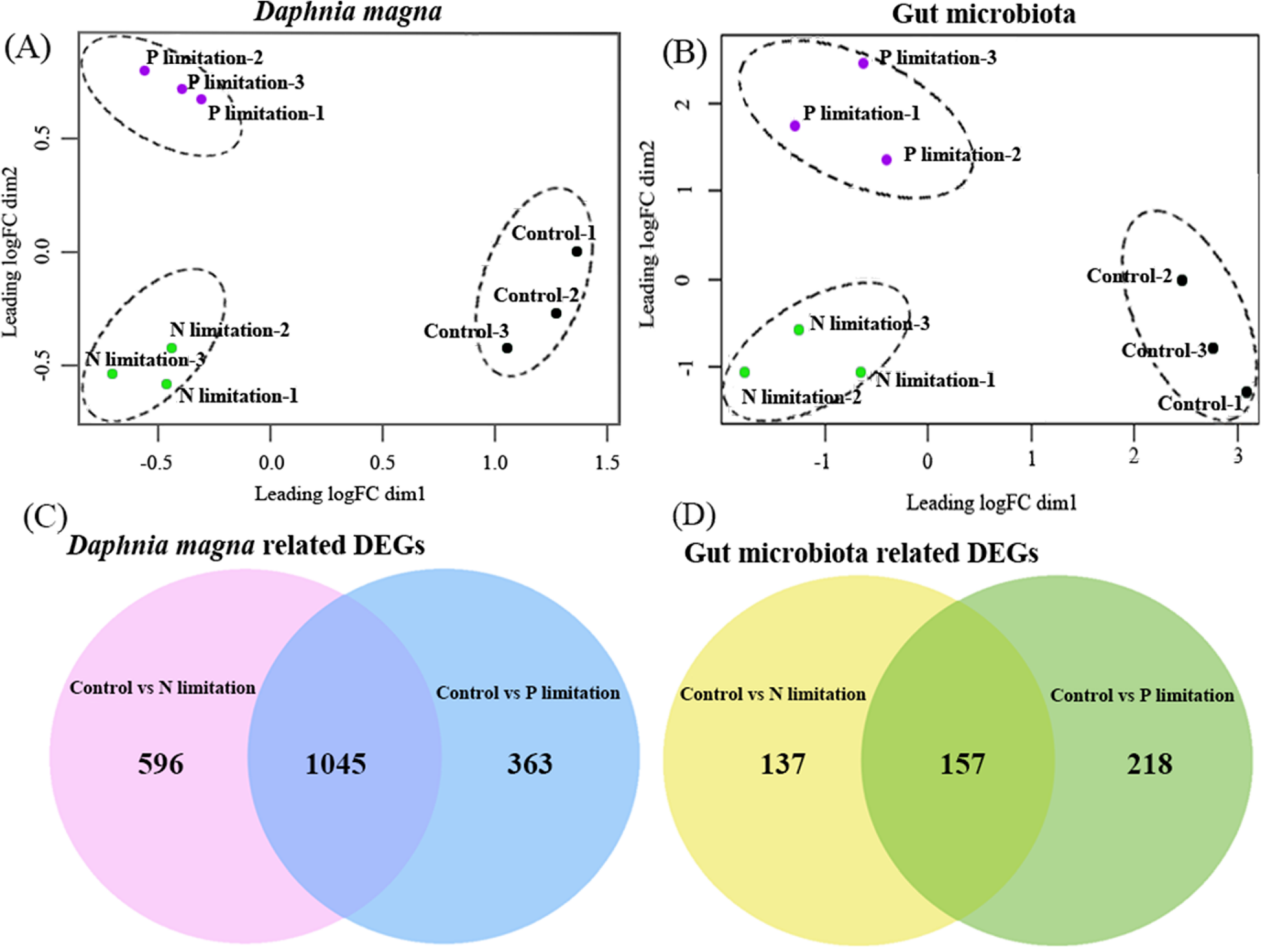
The biological coefficient of variation (BCV) of transcriptomic data affiliated to *Daphnia magna* (*D. magna*) (**a**) and gut microbiota (**b**), using normalized gene expression counts for each experimental group. Venn diagrams showing the number of differentially expressed genes (DEGs) among the different treatment groups for (**c**) *D. magna* and (**d**) gut microbiota

After RNA extraction, as well as sequencing the crushed gut of *D. magna,* the achievement of contamination-free laboratory operations was confirmed by a lack of PCR product in the rinse water. Approximately 131 to 207 million 150 bp paired-end reads were generated across the 9 samples (Table S3). The results showed that after disentanglement of the metatranscriptomic data, the percentage of reads that affiliated to the *D. magna* (host) and gut microbes ranged from 76.92 to 85.03%, and from 7.19 to 35.37%, respectively, across all the samples (Table 2). The number of assembled contigs for the host ranged from 227,920 to 306,664, whereas that for the gut microbiota ranged from 26,418 to 47,344 across all the samples. In addition, the N50 of assembled contigs of the *D. magna* and bacteria ranged from 891 to 1597 (Table S4). A biological coefficient of variation (BCV) result of the identified ORFs in *D. magna* and its gut microbiota indicated that the biological replicates of each experimental group were close, but far from other experimental treatments, which verified significant metabolic differences between different treatments and good repeatability among triplicates (Fig. 3a, b). The result of qPCR is consistent with the RNA sequencing data of gut microbe (Fig. S4) and *D. magna* (Fig. S5), indicating the credibility of the RNA sequencing results. Besides, the RT-qPCR validation of potential DNA contamination showed that there was no signal yielded when the cycle is less than 40 cycles, indicating that there is very little DNA contamination in the extracted RNA.

**Table 2:** Results of sequence disentanglement

Samples	Assigned to host library	Assigned to bacterial library	Shared	Unassigned
Control-1	78.22	13.37	1.64	6.77
Control-2	76.92	11.14	1.52	11.09
Control-3	81.28	9.31	1.74	7.34
N-limitation-1	78.33	10.47	1.75	9.45
N-limitation-2	80.48	9.35	2.10	8.07
N-limitation-3	82.36	7.19	1.96	8.49
P-limitation-1	85.03	6.19	1.68	7.10
P-limitation-2	84.16	6.34	2.21	7.29
P-limitation-3	82.29	6.95	2.44	8.32

All values are % reads from holobiont

### Effects of different types of prey on the gut microbiota community

**Fig. 4:**
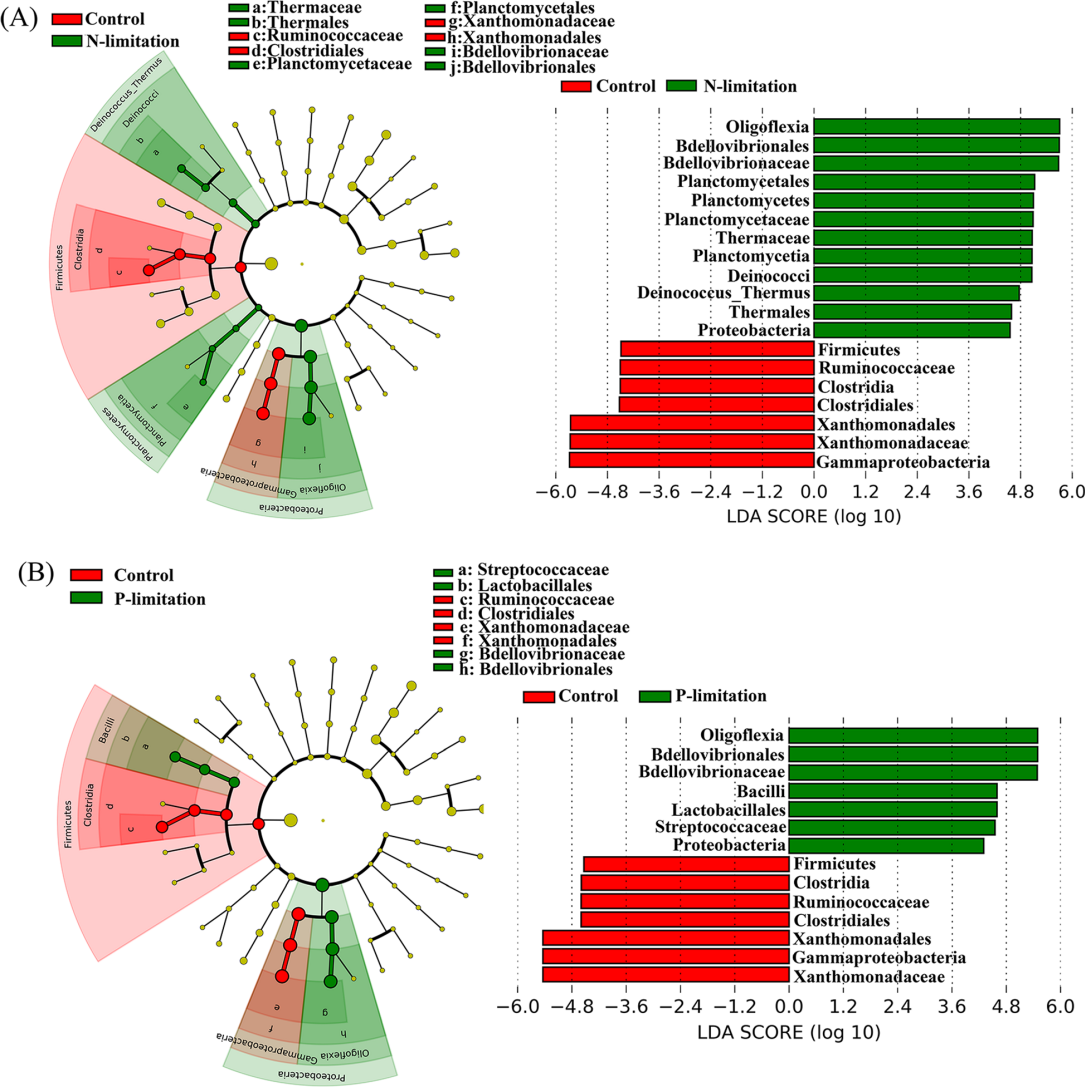
Cladogram indicating the phylogenetic distribution of the microbial lineages in the different experimental treatment groups. The linear discriminant analysis (LDA) score of the taxa representing the odds of their distribution among the comparison of control versus N limitation (**a**) and control versus P limitation (**b**)

Using the amplified and normalized 16S rRNA gene, we identified the taxonomic profiling of the gut microbiota. Our results showed that *Ruminococcaceae* (affiliated to the *Clostridia* class within the *Firmicutes* phylum) and *Xanthomonadaceae*, (affiliated to *Gammaproteobacteria* within the *Proteobacteria*) were significantly enriched in the nutrient-balanced group with LDA scores of 5.76 and 5.43 when compared with the N- and P-limited groups, respectively. Also, *Streptococcaceae* were more abundant in the P-limited groups with LDA scores of 4.7 when compared with the nutrient-balanced group, and *Planctomycetaceae* were enriched in the N-limited group with LDA scores of 4.9 (Fig. 4).

**Fig. 5:**
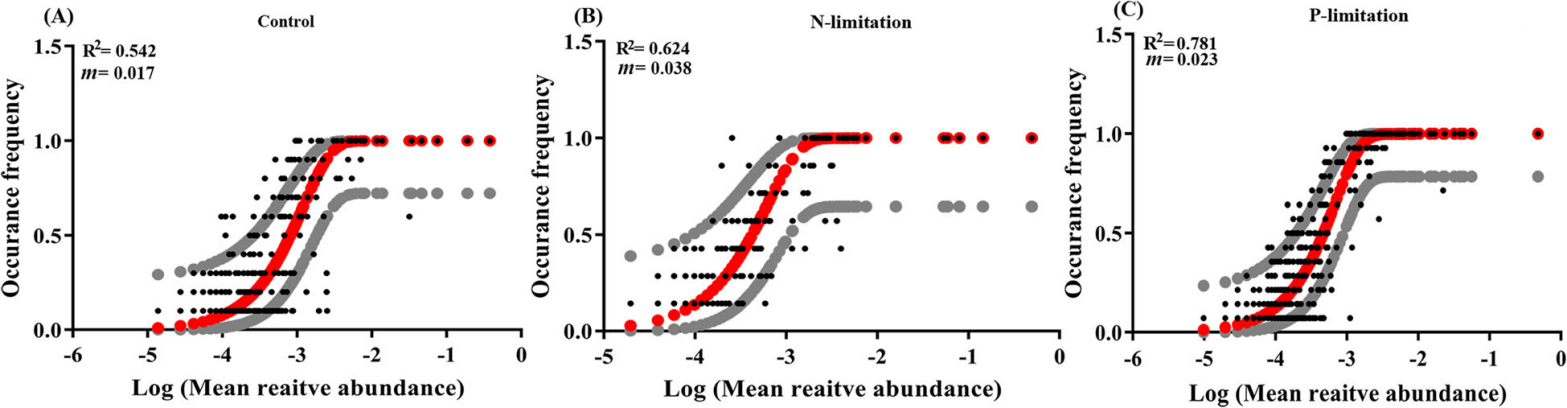
Fit of the neutral model for gut microbial community. **a** Gut microbial community in the Control experimental group. **b** Gut microbial community in the N limitation experimental group. **c** Gut microbial community in the P limitation experimental group. Grey lines represent 95% confidence intervals around the model prediction (solid red line). R^2^ indicates the fit to the neutral model, and *m* indicates the immigration rate

The NCM successfully described the frequency distributions of the 29 gut microbial communities in the Control (*R*^*2*^ = 0.542, *m* = 0.017), N limitation (*R*^*2*^ = 0.624, *m* = 0.038), and P limitation (*R*^*2*^ = 0.781, *m* = 0.023, Fig. 5) diets. A higher R^2^ value in NCM not only indicates a better fit of the model for the microbial community data but also suggests higher importance of neutral process in shaping the community.

### The metabolic variation of gut microbiota under different diets

**Fig. 6:**
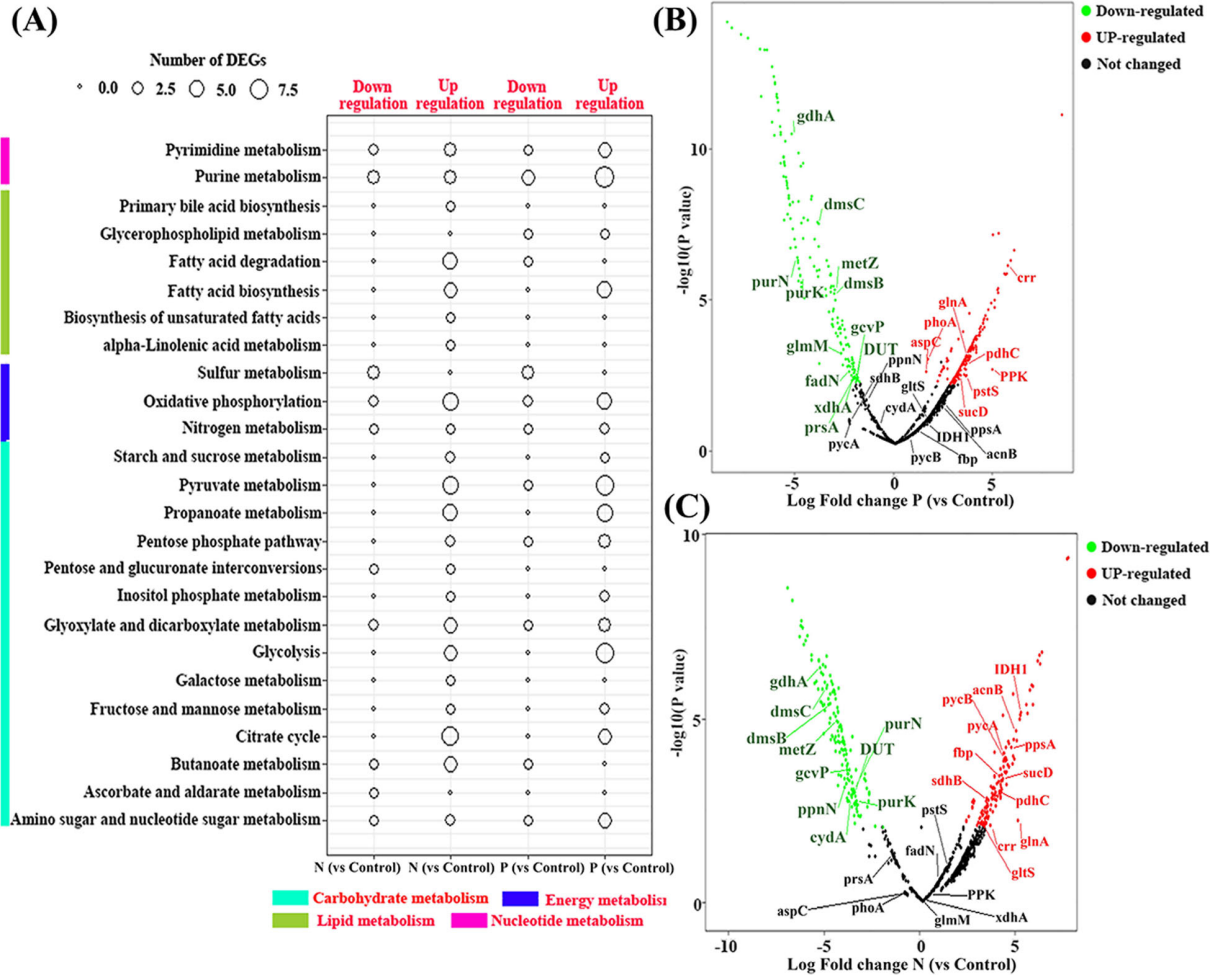
**a** The differentially expressed genes (DEGs) of gut microbiota within different comparison groups at the KEGG module level. DEGs of gut microbiota at gene level in P-limited diet (**b**) and N-limited diet (**c**) with important items highlighted. The important items (i.e. the short fatty acid synthesis, ATP synthesis, nitrogen and phosphorus assimilation, and polyphosphate synthesis related genes): acnB (K01682): aconitate hydratase 2 / 2-methylisocitrate dehydratase; aspC (K00813): aspartate aminotransferase; crr (K02777): PTS system, glucose-specific IIA component; cydA (K00425): cytochrome d ubiquinol oxidase subunit I; dmsB (K00184): dimethyl sulfoxide reductase iron-sulfur subunit; dmsC (K00185): dimethyl sulfoxide reductase membrane subunit; DUT (K01520): dUTP pyrophosphatase; fadN (K07516): 3-hydroxyacyl-CoA dehydrogenase; fbp (K03841): fructose-1,6-bisphosphatase I; gcvP (K00281): glycine dehydrogenase; gdhA (K00261): glutamate dehydrogenase (NAD(P)+); glmM (K03431): phosphoglucosamine mutase; glnA (K01915): glutamine synthetase; gltS (K00284): glutamate synthase (ferredoxin); IDH1(K00031): isocitrate dehydrogenase; metZ (K10764): O-succinylhomoserine sulfhydrylase; pdhC (K00627): pyruvate dehydrogenase E2 component (dihydrolipoamide acetyltransferase); phoA (K01077): alkaline phosphatase; PPK (K00937): polyphosphate kinase; ppnN (K06966): uncharacterized protein; ppsA (K01007): pyruvate, water dikinase; prsA (K00948): ribose-phosphate pyrophosphokinase; pstS (K02040): phosphate transport system substrate-binding protein; purK (K01589): 5-(carboxyamino) imidazole ribonucleotide synthase; purN (K11175): phosphoribosylglycinamide formyltransferase 1; pycA (K01959): pyruvate carboxylase subunit A; pycB (K01960): pyruvate carboxylase subunit B; sdhB (K00240): succinate dehydrogenase / fumarate reductase, iron-sulfur subunit; sucD (K01902): succinyl-CoA synthetase alpha subunit; xdhA (K13481): xanthine dehydrogenase small subunit

The DEGs of gut microbiota are summarized in Table S5. At the KEGG module level, more up-regulated genes were found in the metabolic modules of citrate cycle, glycolysis, propanoate metabolism, and pyruvate metabolism in both N and P limitation experiments (Fig. 6a). Within the energy metabolism category, more up-regulated genes were found in the modules of oxidative phosphorylation (6 for N-limitation, 5 for P-limitation) in both N and P limitation as compared with the Control group. Noticeably, both N and P limitation exhibited more down-regulated genes in the sulfur metabolism module (3 for both P-limitation and N-limitation) than the Control group.

At the gene level, N and P limitation had dramatically influenced the expression pattern of nutrient metabolism-related genes in the gut microbes. For example, compared to the Control group, the phosphate metabolism-related genes polyphosphate kinase (*PPK*), alkaline phosphatase (*phoA*), phosphate transport system (*pstS*), aspartate aminotransferase (*aspC*), and dihydrolipoamide acetyltransferase (*pdhC*) were all up-regulated in P-limited diet, while all these genes were down-regulated in the N-limited diet (Fig. 6b, c). As the gene encoding for glutamine synthetase (*glnA*) plays an important role in both N and P assimilation in bacteria, it was found to be up-regulated in both the N- and P-limited diet. In addition, the previously mentioned down-regulated genes within sulfur metabolism in both treatments were all encoding for anaerobic dimethyl sulfoxide reductase (*dmsC* and *dmsB*). Similarly, the anaerobic fermentation related genes (*gdhA*) were also down regulated in both treatments (Fig. 6b, c). Moreover, after detection of the bacterial polyP in fecal pellets, algal culture, and zooplankton culture under different treatments, the result clearly showed that there is a significantly higher value (*p* < 0.01) of the polyP concentration (normolized by bacterial protein) in the fecal pellets in P-limitation compared to the Control or N-limitation (Table S6).

### The metabolic response of *D. magna* under different diets

**Fig. 7:**
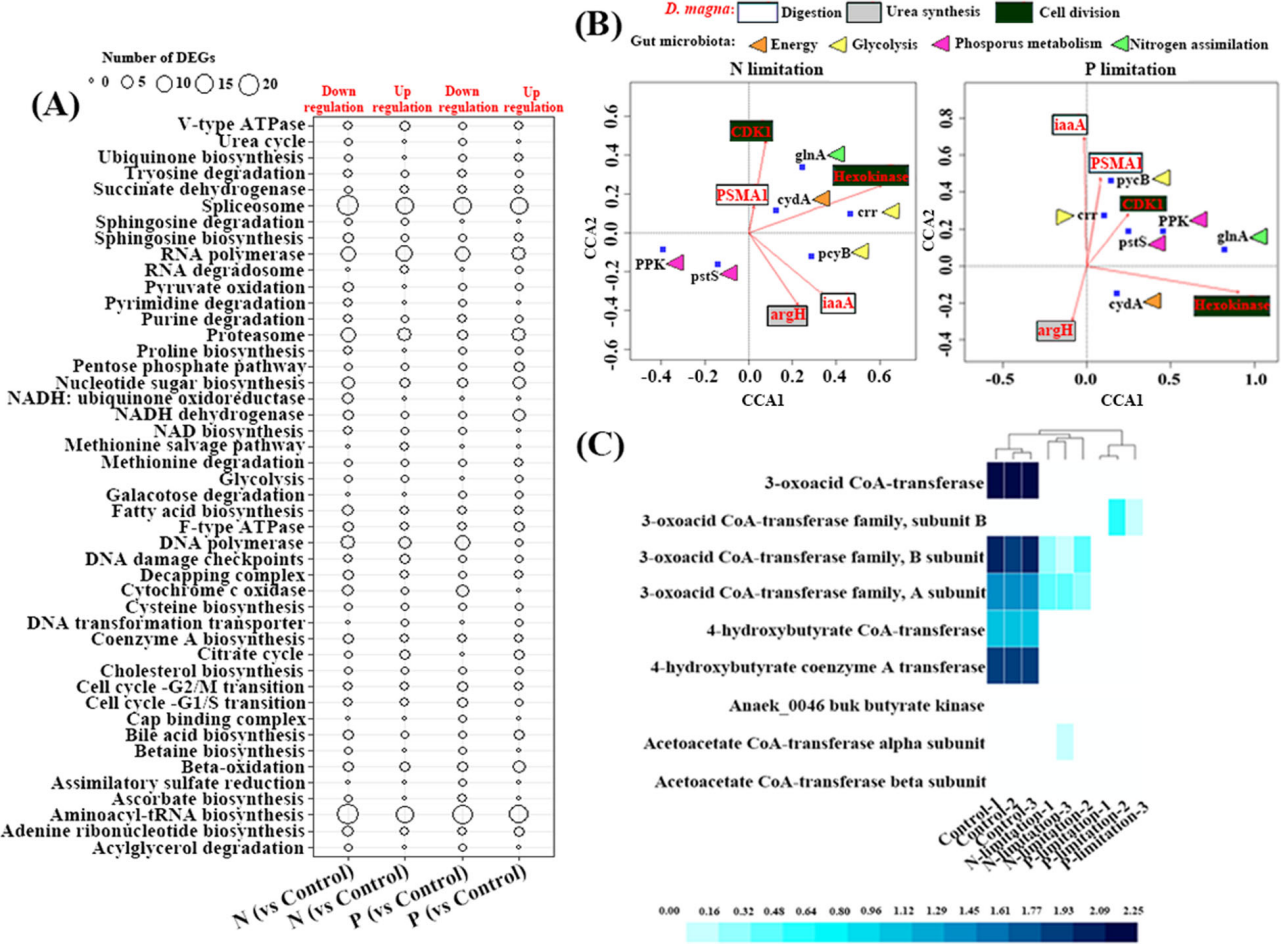
**a** Differentially expressed genes in the *D. magna* among different experimental groups at module level. **b** Canonical correlation analysis (CCA) of the expression level of essential genes between *D. magna* and its gut microbiota. **c** Expression level of microbial genes involved in butyrate synthesis among the different experimental groups

The analysis of *D. magna* affiliated genes had revealed that the nutrient-limited diets mainly affected the energy produce, digestion, and cell replication related genes when compared with the nutrient replete diet. For instance, at the KEGG module level, there are more DEGs enriched in cell replication related spliceosome, Nucleotide sugar biosynthesis, RNA polymerase, DNA polymerase, and Aminoacyl-tRNA biosynthesis; energy produces associated pyruvate oxidation, F-type ATPase, and Cytochrome C oxidase; digestion category affiliated glycolysis, proteasome, and Beta-oxidation modules (Fig. 7a). In addition, genes affiliated to the immune system of *D. magna* showed differences across the treatments (Fig. S3). The result showed that genes affiliated to the KEGG modules, ‘defense response to the bacterium’ and ‘antimicrobial humoral response’, were significantly up-regulated in the P-limited group and down-regulated in the N-limited group when compared with the nutrient-balanced group. In contrast, the genes involved in the ‘negative regulation of defense to the bacterium’ were up-regulated and down-regulated in N- and P-limited groups, respectively when compared with the nutrient-balanced group.

Using CCA, the putative associations of the main differentially expressed genes among the gut microbes and *D. magna* were revealed (Fig. 7b). It is interesting to find that the expression level of gut microbes related genes encoding for phosphorus metabolism (*PPK* and *pstS*) and *D. magna* affiliated genes encoding for digestion were positively correlated in the P-limited diet, but the correlation was negative in the N-limited diet. The glycolysis related microbial genes were positively correlated with the host-associated genes for digestion and cell division in both the N- and P-limited diet. In addition, the microbial expression level for the biosynthesis of the host beneficial representative short-chain fatty acid (SCFA), butyrate, decreased universally under the nutrient-imbalanced algal diet (Fig. 7c).

## Discussion

**Fig. 8:**
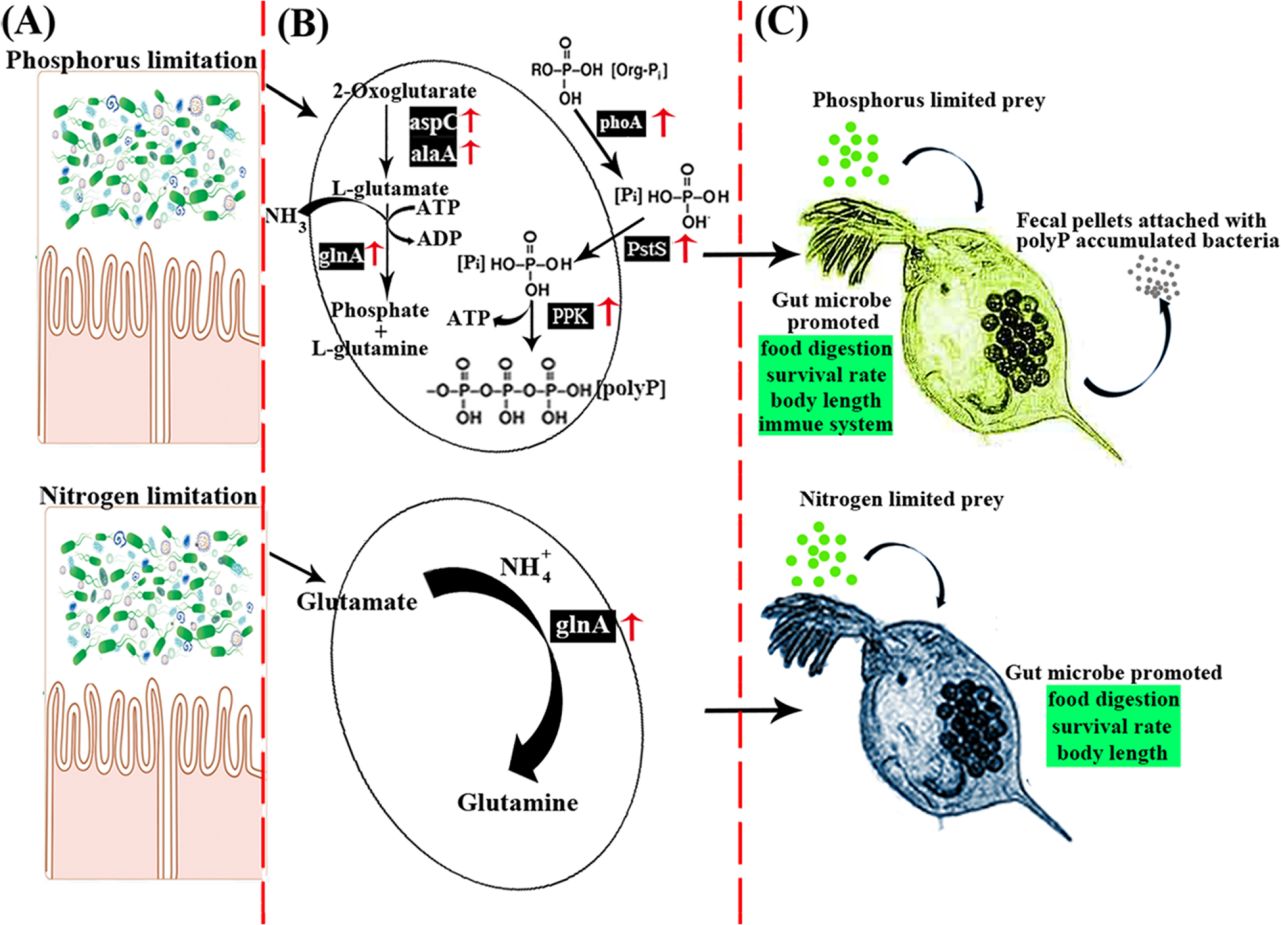
Schematic representation of the main biological pathways in the gut microbiota affected by low quality food. **a** The nutrient-limited algal prey enters into the gut of zooplankton. **b** The major finding of bacterial metabolic reactions towards the nutrient-limited prey. Ingestion of phosphorus-limited prey led to a stimulated accumulation of microbial polyP in the zooplankton gut, whereas ingestion of nitrogen-limited prey promoted nitrogen assimilation metabolism in the intestinal microbiota. **c** The major findings about the effects of nutrient limited algal prey on zooplankton

In this study, we demonstrated for the first time the effects of nutrient-imbalanced prey and environmental nutrient limitation on the interdependence and interplay between the zooplankton *D. magna* and its gut microbiota. In previous studies [6, 20], the researchers mainly focused on the effects of P- and N-limited prey on zooplankton grazing and proliferation. Here, we further demonstrated that the intestinal microbiota not only help the *D. magna* to adapt to the nutrient-imbalanced prey, but they also absorb outstripped nutrients in response to a sudden rise in the level of nutrients from the oligotrophic ambient water to the nutrient-enriched gut (Fig. 8). As the nutrient content of fecal pellets was also promoted by the attachment of nutrient-accumulated intestinal microbes, it is reasonable to believe that these pellets play a more important role in oligotrophic aquatic systems than previously thought.

### Variations in the intestinal microbial community in nutrient-imbalanced algal diet

Living with a nutrient-imbalanced diet markedly altered the microbial community structure in the *D. magna* intestine. The Control group was characterized by enriched levels of *Ruminococcaceae* and *Xanthomonadaceae*. These bacterial families are widely distributed in the gut of metazoans [18, 59], and they are especially effective at degrading a diverse range of polysaccharides and fibers in the gut of wood-feeding metazoans [15, 33]. The high expression level of microbial SCFA synthesis genes we observed in the Control group is consistent with an enriched amount of *Ruminococcaceae* since the bacteria species affiliated to this family is known to be a vital SCFA producer in the gut of metazoan [15]. As the gut microbe-related digestion of polysaccharides and synthesis of SCFA are both essential to the host [35, 60], the compensatory feeding behavior and decreased reproduction ability of *D. magna* in the N- and P-limited groups might be due to the relative decrease of SCFA synthesis- and food digestion-related microbial taxa.

*Planctomycetaceae* were enriched in the gut of the N-limited group. This might be due to the effects of decreased microbial synthesis of butyrate in the gut (Fig. 6b) since it has previously been reported that *Planctomycetaceae* are more abundant in guts containing lower levels of butyrate [77]. Both the P- and N-limited groups had more *Streptococcaceae* in their gut; indeed, previous reports indicate that the presence of *Streptococcaceae* in the gut is highly associated with metabolic disorders of the host [1, 58].

As the mechanisms that control microbial community diversity become an intriguing question to ecologists, the relative importance of selective processes (niche-based or deterministic factors based selection of microbial community) and neutral processes (passive dispersal and ecological drift based stochastic shift of microbial community) have been widely quantified to reveal the driving force of community structure and succession [13, 52]. To better understand whether *D. magna* subjectively selected the gut bacteria to benefit itself in nutrient-limiting conditions, we performed NCM to quantify the importance of neutral processes. The promoted dispersal/immigration rate, *m* value, in N- and P-limitied conditions indicated that the community forms and develops through enhanced immigration from ambient water to the gut. Our result confirmed that the processes of passive dispersal and ecological drift (a neutral process) had an important impact on the distribution of gut microbial communities in all three experimental groups, and the N- and P-limited conditions further increased the importance of the immigration process in shaping *D. magna*’s gut microbial community. The high R^2^ value calculated by the model indicates that the neutral process is the main driving force that shapes the gut microbiome in different diets. When compared with the Control group, the increased R^2^ and *m* value suggest a promoted importance of migration process (i.e. the migration of bacteria from ambient water to the gut of *D. magna*) in shaping the gut microbial community under the low-quality diets [8, 65]. This result, in turn, demonstrated a decreased *D. magna* related influence and increased ambient-gut exchange related influence on its gut microbial community in nutrient-depleted environments. Since zooplankton would ingest more prey under a low-quality diet to compensate for the imbalanced nutrient availability [46, 70], more bacteria could enter the gut. Therefore, the compensatory feeding of *D. magna* may stimulate the migration of bacteria from ambient water to the gut, which in turn reduces the importance of the selection process in low-quality diets. Furthermore, in both treatments, the down-regulated genes in anaerobic sulfur metabolism and fermentation in gut microbes could be also explained by the enhanced ingestion activity of the host. As the increased water filtering activity can lead to the promotion of the oxygen level in the zooplankton gut, the anaerobic sulfur, and formation related metabolism could be strongly affected by it. However, this explanation still needs further verification through field experiments.

### P-limitation stimulates accumulation of microbial polyP in the *D. magna* gut

The grazing activity of zooplankton is known to result in an enrichment of particular nutrients in their gut [71, 72]. Our new analysis revealed that intestinal microbiota are strongly affected by the nutrient-rich gut environment. Our results showed that the microbial pathways involved in the accumulation of polyP were markedly up-regulated in the P-limited group when compared with the Control group, and this was further verified through the detection of microbial polyP inside the fecal pellets produced by the zooplankton. By comparing the concentration of microbial polyP in the algal prey-associated bacteria and the free-living bacteria in *D. magna* culture medium with that of the fecal pellet-associated bacteria, we confirmed that the microbial accumulation of polyP had occurred inside *D. magna* intestine. These results are consistent with previous reports, which demonstrated that bacteria can exhibit rapid and extensive polyP accumulation once inorganic P (Pi) is added to cells that were previously subjected to Pi starvation stress [29, 32]. Therefore, the nutrient-enriched environment of the *D. magna* gut provided the bacteria with excessive levels of Pi without competition from other ambient organisms. Since zooplankton can promote bacterial community changes in the surrounding seawater through farming and nutrient enrichment [64], our new findings suggest that microbial activity in the zooplankton gut might play a more important role than was originally thought in regulating the regeneration of nutrients in oligotrophic aquatic ecosystems.

### N-limitation stimulates microbial nitrogen assimilation in the *D. magna* gut

Our results indicated an increased level of expression of microbial inorganic nitrogen assimilation-related genes in the N-limited group. This suggests that the microbiota that are ingested might utilize ammonia generated by the zooplankton [16] to compensate for their previous nitrogen starvation in ambient water. Considering the strong competition between different microorganisms and the dilution effects of ammonia once it is excreted [27, 66], the intestinal bacteria seem to have a better supply of nitrogen than those living outside the gut. Since some of the ingested microbes are released into the ambient water through fecal pellets [55, 73], utilization of the *D. magna* excreted nitrogen source (ammonia) during their passage through the gut might enhance their physiological fitness in nitrogen-limited environments.

### Effects of nutrient limitation on the cooperation between the gut microbiota and *D. magna* in nutrient provision

Our sterile feeding experiment showed that when the food quality is poor, the existing gut microbiota can still benefit the *D. magna* by enhancing their growth and survival, rather than solely competing for the nutrients that are lacking. The positive correlation between host essential genes for survival (digestion and growth) and gut microbial genes for glycolysis also illustrated that the gut microbe could help the host in food digestion and growth under nutrient-limited conditions. These results are consistent with those from previous reports, which indicate that gut microbiota are essential for the growth and survival of zooplankton under different environmental conditions [10, 43]. We also discovered that the expression level of antimicrobial genes in the host immune system was increased in the P-limited group. This might be explained by the high requirement of phosphorus by zooplankton as compared to bacteria and phytoplankton since phosphorus is essential for egg production of zooplankton [75]. Therefore, the potential nutrient competition between the gut microbiota and their host may lead to the promoted expression of zooplankton immune system affiliated genes for acquiring more phosphorus. For example, it has been reported that phosphorus-limited prey are more damaging to zooplankton than nitrogen-limited prey with decreased body length and neonates production [3, 20]. It is interesting to find that the body length and survival rate of *D. magna* were promoted when compared with the germ-free P-limited group, which could be due to the promoted digestion and absorption capability of *D. magna* when the gut microbiota are present [9, 10]. Therefore, we suggest that by entering the gut of *D. magna*, bacteria not only benefit themselves by absorbing more nutrients inside the gut of their host but also benefit the host by improving the growth of the host in a nutrient-imbalanced algal diet.

## Conclusion

In summary, a metatranscriptomic study of the effects of nutrient-imbalanced algal diets on the metabolism and community composition of *D. magna*’s gut microbiota revealed that P- and N-limited prey promoted polyP accumulation in fecal pellets and nitrogen assimilation in the gut microbiota, respectively. The NCM results suggested that under nutrient-limited conditions, the influence of the host in selecting the gut microbial community was reduced, while the passive dispersal processes were promoted possibly through compensatory feeding. The result of the 16 s rRNA amplicon sequencing indicated that *Streptococcaceae*, which might be responsible for the metabolic disorder of the host, was more abundant in the N-limited and P-limited groups, while *Ruminococcaceae*, known to be a vital SCFA producer in the metazoan gut, was more abundant in the Control group. A nearly axenic grazing experiment demonstrated that the microbiota inside the gut of *D. magna* not only benefited from the nutrient-rich gut environment, but they also helped *D. magna* to achieve better growth in a low-quality diet. Altogether, our study, for the first time, revealed that there is an increased chance for ambient bacteria to enter *D. magna*’s gut under nutrient-limited conditions, and these ingested bacteria can absorb excess nutrients and benefit the growth of their zooplankton host at the same time.

## Supplementary Information

**Additional file 1: Table S1.** Primers for qPCR detection. **Table S2.** The summary of flow cytometer detection of bacteria in sterile algal culture. **Table S3.** Illumina sequencing statistics of mRNA dataset. **Table S4.** Summary of the mRNA assembly and coding regions. **Table S6.** Comparison of bacterial polyphosphate in different experimental groups. **Figure S1.** (A) Agar plate without adding antibiotic cocktail. (B) Agar plate with antibiotic cocktail added. **Figure S2.** (A) Body length of *Daphnia magna* at the end of 7 days, calculated from 13 individuals that survived in Gem-free Control group, 7 individuals that survived in Gem-free N-limitation group, and 9 individuals that survived in Gem-free P-limitation group. (B) Mortality rate of *D. magna* over a period of 7 days, calculated from the triplicates (three 150 mL bottles with 30 individuals in each bottle) of each experimental group. **Figure S3.** The response of immune system in *Daphnia magna* (*D. magna*) under different diets. **Figure S4.** Spearman's correlation between qPCR and RNA sequencing results for the five selected microbial genes. Each point represents a value of fold change. Fold change values were log2 transformed. **Figure S5.** The qPCR verification of selected DEGs of *Daphnia magna* (*D. magna*). MCM2: DNA replication licensing factor MCM2; LMAN2: Vesicular integral-membrane protein VIP36; PDIA3: Protein disulfide-isomerase A3, also known as glucose-regulated protein, 58-kD (GRP58); CTH: Cystathionine gamma-lyase; DNAJC3: DnaJ homolog subfamily C member 3; ahcY: Adenosylhomocysteinase; metK: S-adenosylmethionine synthase.

**Additional file 2: Table S5**.

## Data Availability

Sequence data was deposited in GenBank (Sequence Read Archive) and is available under the BioProject PRJNA597965.

## Abbreviations

LDA: Linear discriminant analysis
LEfSe: Linear discriminant analysis effective size
ORFs: Open reading frames
KEGG: Kyoto Encyclopedia of Genes and Genomes
DEGs: Differentially expressed genes
CCA: Canonical correspondence analysis
BCV: Biological coefficient of variation
SCFA: Short-chain fatty acid
Pi: Inorganic Phosphate
