# Supplementary material for: Nutrient-imbalanced conditions shift the interplay between zooplankton and gut microbiota
Source: BMC Genomics. 2021 Jan 7;22:37. doi: 10.1186/s12864-020-07333-z (PMC7791863; doi:10.1186/s12864-020-07333-z)
Supplement: Supplementary file 1 — Additional file 1: Table S1. Primers for qPCR detection. Table S2. The summary of flow cytometer detection of bacteria in sterile algal culture. Table S3. Illumina sequencing statistics of mRNA dataset. Table S4. Summary of the mRNA assembly and coding regions. Table S6. Comparison of bacterial polyphosphate in different experimental groups. Figure S1. (A) Agar plate without adding antibiotic cocktail. (B) Agar plate with antibiotic cocktail added. Figure S2. (A) Body length of Daphnia magna at the end of 7 days, calculated from 13 individuals that survived in Gem-free Control group, 7 individuals that survived in Gem-free N-limitation group, and 9 individuals that survived in Gem-free P-limitation group. (B) Mortality rate of D. magna over a period of 7 days, calculated from the triplicates (three 150 mL bottles with 30 individuals in each bottle) of each experimental group. Figure S3. The response of immune system in Daphnia magna (D. magna) under different diets. Figure S4. Spearman's correlation between qPCR and RNA sequencing results for the five selected microbial genes. Each point represents a value of fold change. Fold change values were log2 transformed. Figure S5. The qPCR verification of selected DEGs of Daphnia magna (D. magna). MCM2: DNA replication licensing factor MCM2; LMAN2: Vesicular integral-membrane protein VIP36; PDIA3: Protein disulfide-isomerase A3, also known as glucose-regulated protein, 58-kD (GRP58); CTH: Cystathionine gamma-lyase; DNAJC3: DnaJ homolog subfamily C member 3; ahcY: Adenosylhomocysteinase; metK: S-adenosylmethionine synthase. [file 12864_2020_7333_MOESM1_ESM.doc]

**Effects of prey in different nutrient quality on interactions between zooplankton and its gut microbiota**

**Yingdong Li^1^, Zhimeng Xu^1,2,3^,** **Hongbin Liu^1,4*^**

^1^Department of Ocean Science, The Hong Kong University of Science and Technology, Clearwater Bay, Kowloon, China

^2^SZU-HKUST Joint PhD Program in Marine Environmental Science, Shenzhen University, Shenzhen, China.

^3^Institute for Advanced Study, Shenzhen University, Shenzhen, China.

^4^Hong Kong Branch of Southern Marine Science & Engineering Guangdong Laboratory, The Hong Kong University of Science and Technology, Hong Kong, China

**Running Title**: Mutual benefit between microbiota and zooplankton

**Keywords:** Metatranscriptomic analysis, Zooplankton, *Daphnia magna*, Gut microbe, stoichiometric homeostasis

***AUTHORS FOR CORRESPONDENCE:** Hongbin Liu, [liuhb@ust.hk](mailto:hmjing@sidsse.ac.cn);

**Table S1** Primers for qPCR detection.

| Gene | Forward (5’-3’) | Reverse (5’-3’) | Affiliation |
| --- | --- | --- | --- |
| *dsrA* (dissimilatory sulfite reductase alpha subunit) | ACSCACTGGAAGCACGCCGG | GTGGMRCCGTGCAKRTTGG | Gut microbiota |
| butyryl-CoA CoA transferase | GCIGAICATTTCACITGGAAYWSITGGCAYATG | CCTGCCTTTGCAATRTCIACRAANGC | Gut microbiota |
| *narG* (nitrate reductase / nitrite oxidoreductase, alpha subunit) | TAYGTSGGGCAGGARAAACTG | CGTAGAAGAAGCTGGTGCTGTT | Gut microbiota |
| *nirK* (nitrite reductase) | TCATGGTGCTGCCGCGKGACGG | GAACTTGCCGGTKGCCCAGAC | Gut microbiota |
| GAPDH (glyceraldehyde 3-phosphate dehydrogenase) | CCTGCCAAGTATGATGACATCAA | AGCCCAGGATGCCCTTTAGT | Gut microbiota |
| 16S rRNA | TCCTACGGGAGGCAGCAGT | GGACTACCAGGGTATCTAATCCTGTT | Gut microbiota |
| metK S-adenosylmethionine synthetase | ATCGCTCTGCTGCTTATG | TTGGATGTGCCGTAATCG | D. magna |
| ahcY adenosylhomocysteinase | GCGTCAGGAATATCAACCA | CAGGCGAAGATGGAGAAC | D. magna |
| CTH cystathionine gamma-lyase | CTACCGTCTTCAATCTGCTA | GCCGAGTTCATCACAATATC | D. magna |
| DNAJC3 DnaJ homolog subfamily C member 3 | GCATCCAGACAACTTCCA | TCCTCGCCATTATCATACTT | D. magna |
| PDIA3 protein disulfide-isomerase A3 | CAGTCCTTCACCGATTCTT | CAACAGCAGTCTTCAATGG | D. magna |
| LMAN2 lectin, mannose-binding 2 | ATGCTCGTGATAGAATGGTT | ATAGTATCCTGTCGGTAATCG | D. magna |
| MCM2 DNA replication licensing factor MCM2 | GCAAGGCTATTCAGTAAGTG | GTCTCAACGAACGGATGT | D. magna |

**Table S2** The summary of flow cytometer detection of bacteria in sterile algal culture.

| Samples | events/ 100 µL  Before grazing experiment  (Mean ± SD) | events/ 100 µL  After grazing experimen  (Mean ± SD) |
| --- | --- | --- |
| Control_Gem-free | 278±54 | 381±37 |
| N_Gem-free | 315±31 | 414±41 |
| P_Gem-free | 292±42 | 219±32 |

Mean: mean value for different elemental ratio

SD: standard deviation

**Table S3 Illumina sequencing statistics of mRNA dataset.**

| Sample | Base number (Gb) | Raw reads | Clean reads | %>Q30 |
| --- | --- | --- | --- | --- |
| Control-1 | 31.4 | 178776978 | 178727870 | 93.7% |
| Control-2 | 27.6 | 156749216 | 156706280 | 94.1% |
| Control-3 | 35.4 | 207102350 | 207045534 | 93.2% |
| N-limitation-1 | 32.2 | 188350584 | 188297762 | 91.1% |
| N-limitation-2 | 30.3 | 176307328 | 176258290 | 92.5% |
| N-limitation-3 | 32.7 | 185635678 | 185583964 | 92.9% |
| P-limitation-1 | 34.2 | 200718860 | 200663658 | 91.6% |
| P-limitation-2 | 28.7 | 161130766 | 161086154 | 92.1% |
| P-limitation-3 | 23.7 | 131320774 | 131298448 | 90.5% |

**Table S4** Summary of the mRNA assembly and coding regions.

| Sample | Assembled contigs >200  (*Daphnia*) | N50  (*Daphnia*) | Assembled contigs  (Gut bacteria) | N50  (Gut bacteria) | Coding regions (*Daphnia*) | Coding regions  (Gut bacteria) |
| --- | --- | --- | --- | --- | --- | --- |
| Control-1 | 256783 | 1459 | 47344 | 1342 | 99175 | 11353 |
| Control-2 | 267350 | 1516 | 45471 | 1185 | 106046 | 15635 |
| Control-3 | 235527 | 1557 | 42954 | 1088 | 111486 | 11243 |
| N-limitation-1 | 284626 | 1597 | 31182 | 1341 | 108050 | 9342 |
| N-limitation-2 | 267377 | 1555 | 33427 | 1244 | 105465 | 10432 |
| N-limitation-3 | 261048 | 1549 | 32772 | 1374 | 103774 | 9133 |
| P-limitation-1 | 263938 | 1461 | 28952 | 932 | 104011 | 8654 |
| P-limitation-2 | 306664 | 1513 | 26418 | 912 | 111486 | 7624 |
| P-limitation-3 | 227920 | 1462 | 26982 | 891 | 97226 | 8245 |

**Table S6** Comparison of bacterial polyphosphate in different experimental groups.

| Experimental groups | Relative fluorescence units/mg of bacterial protein  (Mean ± SD)  (fecal pellets attached bacteria) | Relative fluorescence units/mg of bacterial protein  (Mean ± SD)  (bacteria in algal culture) | Relative fluorescence units/mg of bacterial protein  (Mean ± SD)  (bacteria in zooplankton culture) |
| --- | --- | --- | --- |
| Control | 14044±522 | 15091±318 | 11205±567 |
| N-limitation | 18126±334 | 9947±336 | 10278±457 |
| P-limitation | 24278±884 *** | 11385±549 | 9998±534 |

Mean: mean value for different elemental ratio

SD: standard deviation

*** denotes statistically significant (P ˂ 0.05) higher value in number of polyphosphate concentration than in control or N-limitation


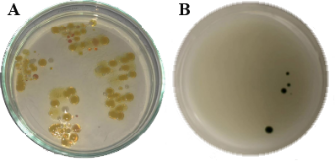


**Figure S1** (A) Agar plate without adding antibiotic cocktail. (B) Agar plate with antibiotic cocktail added.


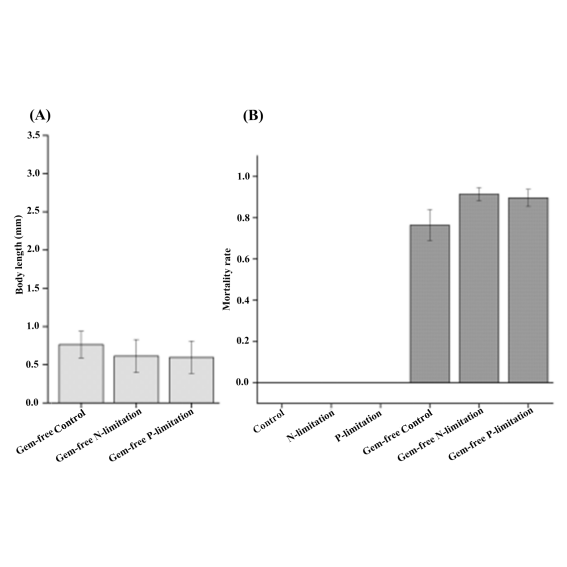


**Figure S2** (A) Body length of *Daphnia magna* at the end of 7 days, calculated from 13 individuals that survived in Gem-free Control group, 7 individuals that survived in Gem-free N-limitation group, and 9 individuals that survived in Gem-free P-limitation group. (B) Mortality rate of *D. magna* over a period of 7 days, calculated from the triplicates (three 150 mL bottles with 30 individuals in each bottle) of each experimental group.


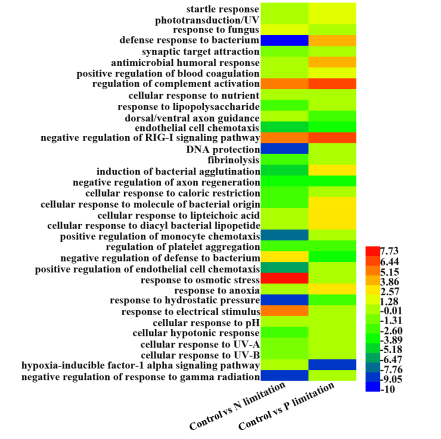


**Fig. S3** The response of immune system in *Daphnia magna* (*D. magna*) under different diets.


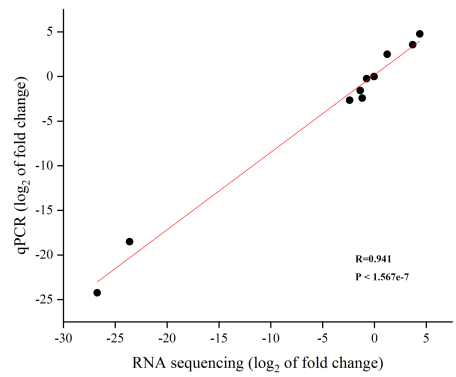


**Fig. S4** Spearman's correlation between qPCR and RNA sequencing results for the five selected microbial genes. Each point represents a value of fold change. Fold change values were log2 transformed.


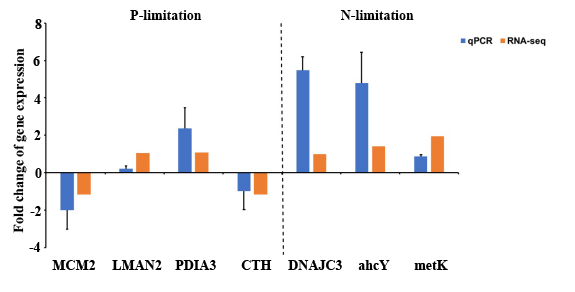


**Fig. S5** The qPCR verification of selected DEGs of *Daphnia magna* (*D. magna*). MCM2: DNA replication licensing factor MCM2; LMAN2: Vesicular integral-membrane protein VIP36; PDIA3:

Protein disulfide-isomerase A3, also known as glucose-regulated protein, 58-kD (GRP58); CTH: Cystathionine gamma-lyase; DNAJC3: DnaJ homolog subfamily C member 3; ahcY: Adenosylhomocysteinase; metK: S-adenosylmethionine synthase.
